# Biohybrid Control of General Linear Systems Using the Adaptive Filter Model of Cerebellum

**DOI:** 10.3389/fnbot.2015.00005

**Published:** 2015-07-20

**Authors:** Emma D. Wilson, Tareq Assaf, Martin J. Pearson, Jonathan M. Rossiter, Paul Dean, Sean R. Anderson, John Porrill

**Affiliations:** ^1^Sheffield Robotics, University of Sheffield, Sheffield, UK; ^2^Bristol Robotics Laboratory (BRL), University of Bristol, Bristol, UK; ^3^Bristol Robotics Laboratory (BRL), University of the West of England, Bristol, UK; ^4^Department of Automatic Control and Systems Engineering, University of Sheffield, Sheffield, UK

**Keywords:** cerebellum, adaptive control, adaptive filter, electroactive polymer, artificial muscles, soft robotics

## Abstract

The adaptive filter model of the cerebellar microcircuit has been successfully applied to biological motor control problems, such as the vestibulo-ocular reflex (VOR), and to sensory processing problems, such as the adaptive cancelation of reafferent noise. It has also been successfully applied to problems in robotics, such as adaptive camera stabilization and sensor noise cancelation. In previous applications to inverse control problems, the algorithm was applied to the velocity control of a plant dominated by viscous and elastic elements. Naive application of the adaptive filter model to the displacement (as opposed to velocity) control of this plant results in unstable learning and control. To be more generally useful in engineering problems, it is essential to remove this restriction to enable the stable control of plants of any order. We address this problem here by developing a biohybrid model reference adaptive control (MRAC) scheme, which stabilizes the control algorithm for strictly proper plants. We evaluate the performance of this novel cerebellar-inspired algorithm with MRAC scheme in the experimental control of a dielectric electroactive polymer, a class of artificial muscle. The results show that the augmented cerebellar algorithm is able to accurately control the displacement response of the artificial muscle. The proposed solution not only greatly extends the practical applicability of the cerebellar-inspired algorithm, but may also shed light on cerebellar involvement in a wider range of biological control tasks.

## 1 Introduction

The cerebellum is a region of the brain strongly associated with adaptive control and skilled movement (Ito, 1984; Dean et al., 2010). Its importance is highlighted by the fact that it contains up to 80% of all neurons in the human brain (Herculano-Houzel, 2010). These cells are arranged in a very uniform way into discrete cerebellar microcircuits that are repeated across the cerebellar cortex (Eccles et al., 1967; Marr, 1969; Albus, 1971). This suggests that there is a single “cerebellar algorithm,” implemented in the control of many different tasks (Ito, 1984, 2008; Porrill et al., 2013) where the control function of each individual region of the cerebellum depends both on this internal algorithm and the architecture in which it is embedded (Lisberger, 1998; Porrill et al., 2013). Understanding this cerebellar algorithm is a fundamental step toward understanding the biological computations involved in sensorimotor control. Moreover, in certain respects, the flexibility, grace, and complexity of biologically controlled movement are superior to its robotic counterpart. The cerebellar algorithm is a natural place to start to investigate the origin of this superiority, with the aim of improving robotic control.

Cerebellar function has been widely modeled using many computational approaches, which can be broadly grouped into the following categories: (i) descriptive models, focusing on neural dynamics, often based on compartmental models (Bower and Beeman, 1994; De Schutter and Bower, 1994; Gleeson et al., 2007); (ii) look-up tables, such as the cerebellar model articulation computer (CMAC) (Albus, 1971), which are now rarely used in neuroscience studies; (iii) olivary models, where cerebellar output is driven by inputs from the inferior olive to the Purkinje cell (Jacobson et al., 2008; Torben-Nielsen et al., 2012); and (iv) Marr–Albus models (Marr, 1969; Albus, 1971), which include many variations (Kawato and Gomi, 1992; Schweighofer et al., 1996; Medina and Mauk, 2000), and in particular, the adaptive filter model of cerebellar function (Fujita, 1982; Dean et al., 2010).

The adaptive filter model is distinctive because it has been analyzed in a variety of simulated biological control problems (Dean et al., 2002; Porrill and Dean, 2007b), sensory processing problems (Anderson et al., 2012; Dean et al., 2013), and neurorobotic tasks (Lenz et al., 2009; Anderson et al., 2010). Furthermore, the adaptive filter model in particular is able to represent both forward and inverse models (Wolpert et al., 1998), and therefore appears to be able to reconcile different proposed functional roles within a single computational description (Porrill et al., 2013). The advantages of this model are: (i) it is a simple homogeneous representation making it easy to implement and analyze; (ii) the learning rule is biologically plausible; (iii) when used in a biologically inspired recurrent architecture, it uses the available sensory error to drive adaptation, rather than the unavailable motor-error signal (Porrill et al., 2004; Porrill and Dean, 2007b; Dean et al., 2010); (iv) the recurrent architecture also leads to increased controller modularity for MIMO systems, allowing separable controllers to be designed in task space rather than motor command space (Porrill et al., 2004; Porrill and Dean, 2007b). These attributes and wide functional usage suggest that the adaptive filter model is not only a leading computational description of cerebellar function, but that analysis of this model is beginning to reveal some of the origins of the superiority of biologically controlled movement.

One limitation of the adaptive filter model of cerebellum is that it has, to date, only been applied in the dynamic control context to problems where mass or inertia are negligible, such as velocity control in eye movements, i.e., the vestibulo ocular reflex (VOR) (Dean et al., 2002; Lenz et al., 2009). In linear systems theory, this type of plant under control (the eye) corresponds to a special class of system (that has an equal number of poles and zeros). The cerebellar algorithm is only applicable at present to this special class, and not to, for instance, position or velocity control in plants with inertia. These types of plant are of a more general class, which are known as strictly proper, i.e., they have more poles than zeros.

Application of the cerebellar algorithm as it stands to strictly proper systems would lead to instabilities in learning and control (because the cerebellar filter would have to learn an improper inverse model of the plant). This means that it cannot deal naturally with, for example, robotic systems involving inertia, such as dynamic control of a robot arm. This constraint severely limits the practical applicability of the algorithm in domains, such as robotics, and also raises the important theoretical question of how the cerebellum handles this type of control problem in biological systems. The aim of this paper, therefore, is to develop a novel control scheme where the adaptive filter model can be applied to the inverse plant control of strictly proper systems. The approach we take is to augment the cerebellar-inspired controller with a model reference adaptive control (MRAC) scheme (Landau, 1979; Kaufman et al., 1998). This approach results in a biohybrid controller, which has the key advantage that it allows us to apply cerebellar-inspired control to a wide class of engineered systems. We will discuss at the end of the paper the implications and relevance of this scheme for biology.

Embodying neural algorithms in physical systems is an important method of evaluating their performance in real world conditions (Webb, 2002; Cuperlier et al., 2007; Kaplan, 2008) and has been widely used to evaluate cerebellar-inspired algorithms (Miller, 1989; van der Smagt, 1998; Spoelstra et al., 1999; Carrillo et al., 2008; Lenz et al., 2009; Anderson et al., 2010). In order to investigate and evaluate the performance of the cerebellar-adaptive filter model with MRAC scheme, we apply it to the displacement control of a dielectric electroactive polymer (DEAP). DEAPs emulate some of the desirable properties of natural muscle: they are capable of producing large strains, have a relatively fast response, and have the potential capacity for self-sensing if an electrical characteristic of the DEAP (such as the capacitance) can be measured (Bar-Cohen, 2001; Pelrine et al., 2002; Xie et al., 2005; OHalloran et al., 2008; Assaf et al., 2013; Gisby et al., 2013). They also present a number of interesting control challenges similar to those posed by natural muscle; they exhibit non-linear dynamics, are manufactured with wide tolerances, and are subjected to creep and time-related aging (Xie et al., 2005). This makes them an excellent test bed for evaluating and furthering our understanding of the cerebellar algorithm. In this contribution, we focus on the performance of the biohybrid algorithm when applied to actuators where the dynamics change over time and between actuators, so we do not consider self-sensing or non-linear control.

The paper is organized as follows. The cerebellar control algorithm, methods of achieving fast learning, and model reference adaptive control (MRAC) extensions are presented in first part of Section 2. Details of the experimental set up, and methods to test the displacement control of a DEAP are given in second half of Section 2. The results are presented in Section 3, and a discussion given in Section 4. Results indicate that the extended cerebellar control algorithm is able to accurately control the displacement response of a DEAP over time and that the MRAC extension provides a technical solution to extending the cerebellar adaptive filter model to the control of strictly proper plants. This extension greatly increases the range of applicability of the algorithm without a substantial increase in design complexity. It therefore represents an important step forward in investigating its use for the more complex non-linear and multivariable systems in which we expect significant advantages of biological control to become apparent.

## 2 Materials and Methods

In this section, the basic adaptive filter model of the cerebellum is first described. Extensions to this algorithm, which include using a reference model (MRAC) are then described. The second part of this section describes how the extended, biohybrid control algorithm was embodied in the real-time control of a dielectric electroactive polymer (DEAP) artificial muscle. We provide a summary of the experimental set-up and the hardware used to test the algorithm, an estimate of a model describing the DEAP dynamics with details on how this model is used to inform the control design, and a summary of the overall control algorithm embodiment.

### 2.1 Basic cerebellar algorithm

**Figure 1:**
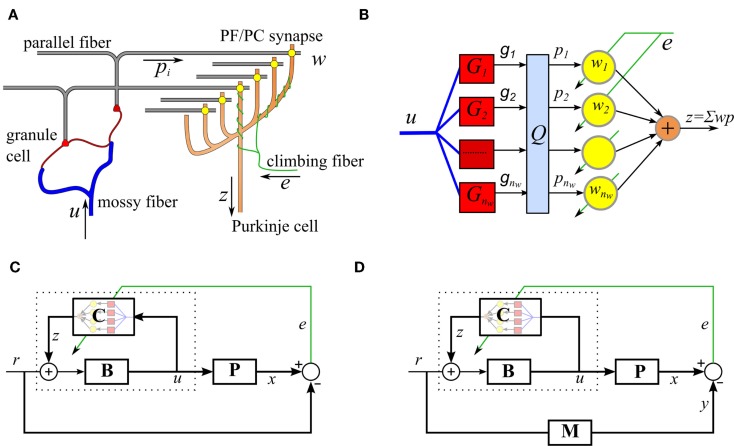
**Simplified cerebellar microcircuit as an adaptive filter**. **(A)** Schematic of the basic cerebellar microcircuit. A mossy-fiber input signal is distributed over many granule cells whose axons form parallel fibers that synapse on Purkinje cells. The climbing fiber signals are assumed to carry error information and act as a teaching signal for the synaptic weights. **(B)** Interpretation of the cerebellar microcircuit as an adaptive filter. Processing by the granule cells is modeled as a bank of time-invariant linear filters, *G_i_*, followed by a fast learning transformation using a fixed matrix, *Q*. The Purkinje cell output takes a weighted sum of the inputs, *p_i_*, where the weights are adjusted using the decorrelation learning rule. **(C)** Model of VOR control, using the recurrent architecture, C represents the adaptive cerebellar filter, B is a fixed brainstem filter, and P the oculomotor plant. **(D)** Cerebellar control architecture with reference model M.

We begin by describing the adaptive filter algorithm that the repeated cerebellar microcircuit is thought to implement. In this section, we restrict ourselves to the case where the reference model is trivial (**M** = 1), so that model reference control reduces to trajectory control. The modifications required to support model reference control will be described in the next section. The cerebellar microcircuit can be modeled as an adaptive filter (Fujita, 1982; Dean et al., 2010), as illustrated in Figures 1A,B. In this model, the Purkinje cell output, *z_t_*, is modeled as a weighted combination of its parallel fiber inputs,

(1) $$z_{t}=\sum_{i=1}^{n_{w}}w_{i,t}p_{i,t}=w_{t}^{T}p_{t}$$

where *n_w_* is the number of weighted parallel fiber connections, *p_i,t_* represents the *i^th^* parallel fiber signal at time *t*, *w_i,t_* is the *i^th^* weight at time *t*, $w_{t}={\left(w_{1,t},\ldots ,w_{n_{w},t}\right)}^{T}$ and $p_{t}={\left(p_{1,t},\ldots ,p_{n_{w},t}\right)}^{T}$.

The adaptive filter weights, *w_t_*, are learnt using the decorrelation learning rule (Sejnowski, 1977), which is identical to the least mean squares (LMS) rule from adaptive control theory (Widrow and Stearns, 1985),

(2) $${\dot{w}}_{t}=-\gamma e_{t}p_{t}$$

where *γ* is a gain that affects the learning rate and *e_t_* is the error signal, where for the basic inverse plant control problem described in Figure 1C, the error signal is defined as

(3) $$e_{t}=r_{t}-x_{t},$$

where *r_t_* is a reference signal and *x_t_* is the plant output.

The parallel fiber signals, ***p**_t_*, are derived from the mossy-fiber inputs to the cerebellum, *u_t_*, after processing by the granule cell layer. Here, the granule cell layer is represented by a bank of time-invariant basis filters, where the filter outputs *g_i,t_* are orthonormalized by a fixed matrix $Q\in R^{n_{w}\times n_{w}}$ in order to increase the rate of learning (Figure 1B),

(4) $$p_{t}=Qg_{t},$$

where $g_{t}={\left(g_{1,t},\ldots ,g_{n_{w},t}\right)}^{T}$. In this investigation, we model the dynamics of the granule cell layer basis filters as a finite number, *n_w_*, of time-invariant second order linear filters, each of the form

(5) $$T_{i}^{2}{\ddot{g}}_{i,t}+2T_{i}{\dot{g}}_{i,t}+g_{i,t}=u_{t},\;\;i=1,\ldots ,n_{w}$$

where *u_t_* is a common mossy-fiber input driving all granule cell basis filters, *T_i_* is the time constant of the *i^th^* basis filter and the dynamics of each filter are set to be critically damped (often referred to as an alpha function). The choice of alpha functions is not critical but does simplify the design because only the single parameter, *T_i_*, must be chosen to define each filter. The choice of alpha functions is also similar to the spectral timing representation suggested by Bullock et al. (1994). Here we choose *T_i_* values to be log-spaced, motivated by coarse coding of time in biological systems, in which signals with larger delay are more dispersed in time, allowing a compact representation of phenomena on both fast and slow time-scales. This biologically inspired basis set provides a computationally efficient low-dimensional basis, which has been shown to outperform conventional basis filters (such as Laguerre bases) on problems involving multiple time scales (Porrill et al., 2009).

The transformation matrix, *Q*, that is used in online processing is designed offline by estimating the brainstem output when there is no cerebellar contribution (*z* = 0) and filtering this brainstem output through the bank of cerebellar alpha filters to give the filter outputs, ***g***_t_, when there is no learning. Samples of ***g***_t_ are combined into a data matrix $\Gamma \in R^{n_{w}\times n_{t}}$, where *n_t_* is the number of data samples (note that for a long batch *n_t_* > *n_w_*),

(6) $$\Gamma =\left[g_{1},g_{2},\ldots ,g_{n_{t}}\right]$$

Using an economy, or thin, singular value decomposition, which retains only the non-zero singular values (Golub and Van Loan, 2012), Γ is expressed as a matrix product

(7) $$\Gamma ={\mathrm{U\Sigma V}}^{T}$$

where the matrices $U\in R^{n_{w}\times n_{w}}$ and $V\in R^{n_{t}\times n_{w}}$ have orthonormal columns and the matrix $\Sigma \in R^{n_{w}\times n_{w}}$ is diagonal (the diagonal elements are the singular values of the data matrix Γ). Here, we assume that Γ is full rank, so that it has *n_w_* non-zero singular values, this will usually be the case for numerical data. However, very small singular values can often be omitted from the decomposition above without losing accuracy, sometimes greatly reducing the size of the transformed bases. An appropriate transformation matrix for fast learning is then given by

(8) $$Q=\Sigma^{-1}U^{T}$$

where the term **U***^T^* orthogonalizes the signals, so that they transmit independent information, and Σ^−1^ normalizes the signals, so that they have equal power. Orthonormalization of filter inputs in this way is commonly used to speed up learning in adaptive signal processing (Haykin, 2001) – the relevance of this orthonormalization procedure to the biological processing in the granule cell layer is considered in the discussion.

The control function of each cerebellar microcircuit also depends on the particular architecture, which it is embedded in. Here, we consider motor plant compensation, as implemented by the vestibular ocular reflex (VOR). A control scheme based on the VOR is shown in Figure 1C. The brainstem **B** is an approximate feedforward controller of the plant **P** and the cerebellar adaptive filter **C** fine tunes this control. The inclusion of a brainstem element is suggested by the observation that the cerebellar pathway is never solely responsible for a behavior, lesions of the cerebellum lead to loss of speed or skill rather than loss of function. In engineering applications, this limits the amount of gain that needs to be stored in the cerebellar component and allows the use of the recurrent connectivity shown in Figure 1. It also supplies a site for learning transfer, a powerful feature of biological systems (not included in the simulations presented here), in which learning in the cerebellar component can be transferred to the brainstem component over time (Porrill and Dean, 2007a).

Exact plant compensation is achieved when the desired cerebellar filter is

(9) $$C^{*}\left(s\right)=B^{-1}\left(s\right)-P\left(s\right)=\sum {w_{i}}^{*}Q_{\mathrm{ij}}G_{j}\left(s\right)$$

where the above equation is given in the Laplace domain and *s* is the Laplace variable, $C^{*}\left(s\right)$ is the transfer function of the ideal cerebellar filter and we assume synaptic weights, ${w_{i}}^{*}$, can be found to satisfy the above equation for the range of **P**(*s*) and **B**^−1^(*s*) under consideration. This assumption requires the basis functions **G***_i_*(*s*) to be sufficiently complete for the given control task. Here, the alpha basis filters in the Laplace-domain are

(10) $$G_{i}\left(s\right)=\frac{1}{{\left(T_{i}s+1\right)}^{2}},\;\;i=1,\ldots ,n_{w}.$$

Plant compensation is achieved using a combination of a fixed feedforward brainstem controller **B**(*s*), and a recurrently connected adaptive element **C**(*s*) (as indicated by dotted lines in Figure 1C),

(11) $$K\left(s\right)=\frac{B\left(s\right)}{1-C\left(s\right)B\left(s\right)}$$

When the cerebellar controller is optimal, the overall optimal controller, $K^{*}\left(s\right)$ will equal the inverse of the plant and is given as

(12) $$K^{*}\left(s\right)=\frac{B\left(s\right)}{1-C^{*}\left(s\right)B\left(s\right)}=P^{-1}\left(s\right)$$

The cerebellar filter **C**(*s*) is embedded in a recurrent loop. Recurrent connectivity is a characteristic feature of the cerebellum (Kelly and Strick, 2003), possibly because in this architecture, output error is a suitable training signal and no prior model of the inverse plant is required (Porrill and Dean, 2007b). The ability to use sensory errors directly as teaching signals greatly simplifies the design of adaptive systems, since it places the focus of design on connectivity (which varies from problem to problem) rather than the learning algorithm, which is the same for each problem (Porrill et al., 2013). This feature may account for the very large range of tasks in which the cerebellum is implicated, and for the flat, homogeneous structure of cerebellar control. It contrasts greatly with the multi-component, task-based approach so characteristic of conventional control design.

### 2.2 Biohybrid adaptive control scheme

If the cerebellar-inspired algorithm is to be generally useful it must be applicable to the strictly proper plants found in many robotic applications. For strictly proper plants, the inverse compensator has an improper transfer function, which is difficult to realize in practice, since it requires differentiators, gives noisy high frequency performance, and can lead to instabilities in the learning rule.

The need to control strictly proper plants is also a feature of many biological motor systems. For example, models of the transfer function of the oculomotor plant usually have one more pole than zeros, so the plant is strictly proper. In some applications, such as eye stabilization by the VOR, we know that the biological system controls velocity rather than position (Dean et al., 2002); this ensures that the overall plant is proper (since the transfer function for velocity gains a factor of *s* and has an equal number of poles and zeros). Our previous work on the adaptive filter model of the cerebellum in the specific context of the VOR (Dean et al., 2002; Porrill et al., 2004; Porrill and Dean, 2007a) used velocity control methods. The oculomotor system can control position directly, but it does so using a special class of fast eye movements known as saccades. It may be that all biological control of strictly proper plants works like this, by supplementing direct control of higher derivatives of position with specialized solutions for achieving partial control of lower derivatives, but at present, we do not have sufficient evidence from biology to propose a strictly biomimetic solution to the problem.

In the absence of specific information as to how biological systems solve this problem, we propose a biohybrid approach in which we incorporate a reference model scheme (Figure 1D) into the cerebellar learning algorithm. This approach is known as model reference adaptive control (MRAC) (Landau, 1979; Kaufman et al., 1998), and is commonly used to define the desired closed loop performance in engineered systems. The behavior of the controlled plant matches that of the reference model **M**, which specifies a realistic response for the controlled plant; the use of a reference model also ensures that the estimated controller is proper (Sastry and Bodson, 1989). When a reference model is used, the ideal controller becomes

(13) $$K^{*}\left(s\right)=\frac{B\left(s\right)}{1-C^{*}\left(s\right)B\left(s\right)}=M\left(s\right)P{\left(s\right)}^{-1}$$

the ideal cerebellar filter is then

(14) $$C^{*}\left(s\right)=B^{-1}\left(s\right)-M^{-1}\left(s\right)P\left(s\right)$$

MRAC is a technical solution, which enables the cerebellar algorithm to function independently of the plant order. MRAC can be applied to the control of general plants of the form,

(15) $$P\left(s\right)=\frac{\prod_{i=1}^{m}s+b_{i}}{\prod_{j=1}^{n}s+a_{j}}$$

where **P**(*s*) is the laplace transform model of a plant with *m* zeros and *n* poles and *n* ≥ *m*. The corresponding reference model is,

(16) $$M\left(s\right)=\frac{1}{{\left(\tau s+1\right)}^{m-n}}$$

where **M**(*s*) specifies the desired response of the controlled system, *τ* is a constant, and (*m* − *n*) the reference model order. The time constant, *τ*, can be chosen, so the response rolls-off at frequencies above the operating range of the plant; so the overall response is not affected much (Kaufman et al., 1998). If the reference model order (*m* − *n*) is large, for improved numerical robustness, the reference model can be implemented as a cascade of first order filters (Proakis and Manolakis, 1988; Anderson et al., 2010),

(17) $$M\left(s\right)=\prod_{i=1}^{m-n}\left(\frac{1}{\tau s+1}\right)$$

Model reference control attempts to minimize the difference between actual output and reference model output

(18) $$e_{t}=y_{t}-x_{t}$$

[replacing equation (3)]. The learning rule, which minimizes the mean squared performance error (*e*^2^), is then

(19) $${\dot{w}}_{t}=-\gamma e_{t}{\bar{p}}_{t}$$

which has a similar form to the learning rule in equation (2), but uses the reference model error defined above, while the parallel fiber signals ${\bar{p}}_{t}={\left({\bar{p}}_{1,t},\ldots ,{\bar{p}}_{n_{w},t}\right)}^{T}$, where ${\bar{p}}_{i,t}=M_{t}*p_{i,t}$ and $M_{t}$ is the reference model in the time domain.

### 2.3 Experimental setup

**Figure 2:**
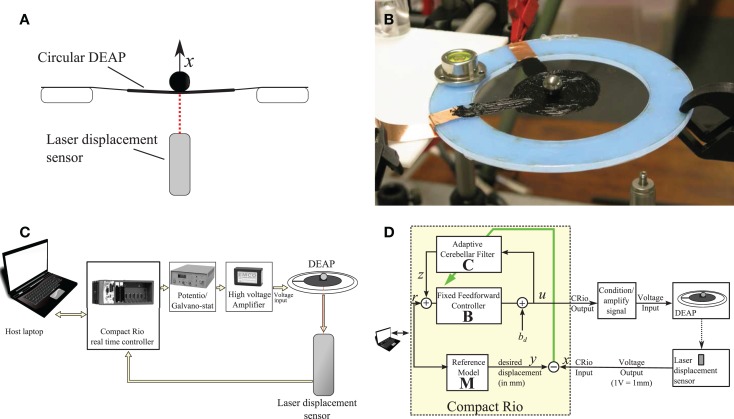
**Summary of experimental set up**. **(A)** A laser sensor is used to measure the vertical displacement of a mass on a circular DEAP. **(B)** DEAP actuator stretched on a circular Perspex frame supporting a spherical load. **(C)** Equipment used during experiments. **(D)** Control system and connectivity for real-time cerebellar control experiments.

In order to investigate and evaluate the cerebellar control scheme with reference model, the scheme was embodied in a real-time control machine for displacement tracking control of a single DEAP actuator. In the experiment, a small spherical mass was loaded on a DEAP and the cerebellar algorithm controlled the vertical displacement of the DEAP. The control scheme had to track a band-limited white noise displacement reference signal. A summary of the experimental set up is given in Figure 2 and details of the experiment are provided below.

The biohybrid control algorithm (Table 1) was implemented in LabVIEW and from there embodied in a CompactRio (CRIO-9014, National Instruments) platform, with input module NI-9144 (National Instruments) and output module NI-9264 (National Instruments) used in combination with a host laptop computer. LabVIEW was run on the host laptop computer, with communication between the host laptop and CompactRio (CRio) carried out using the LabVIEW shared variable engine (Figure 2C). This real-time setup enabled accurate timing of control inputs and outputs. In all experiments, all signals were sampled simultaneously with a sampling frequency of 50 Hz.

**Table 1:** **Biohybrid adaptive control algorithm**.

	Biohybrid algorithm	For each time step, k
1	*y_k_* = **M**(*q*)*r_k_*	Filter input signal through reference model **M**
2	*g_i_*,*_k_* = **G***_i_*(*q*)*u_k−1_*	Filter motor command through bank of *i* alpha basis filters
3	***p****_k_* = Q***g**_k_*	Orthonormalize parallel fiber signals via SVD for fast learning
4	$z_{k}=w_{k}^{T}p_{k}$	Adaptive filter output
5	*v_k_* = **B**(*q*)(*r_k_* + *z_k_*)	Filter input signal and adaptive filter output through brainstem **B**
6	*u_k_* = *v_k_* + *b_d_*	Add discrete offset onto brainstem output to give motor command
7	write *u_k_* to CRio output	Motor command used to drive DEAP actuator
8	read *x_k_* from CRio input	Measure DEAP displacement using laser displacement sensor
9	*e_k_* = *x_k_* − *y_k_*	Error calculation
10	${\bar{p}}_{k}=M\left(q\right)p_{k}$	Filter parallel fiber signal through reference model
11	$w_{k+1}=w_{k}-\beta e_{k}{\bar{p}}_{k}$	Update adaptive filter weights (where *β* = *γdt*)

*This extended cerebellar algorithm with reference model corresponds to the architecture given in Figure 1D. Discrete-time filters are used in the implementation, where M(*q*) is the discrete version of the continuous filter M(*s*) calculated using zero-order-hold and *q* is the shift operator (*qu_k_* = *u_k + 1_*)*.

Regarding design parameters of the cerebellar filter **C**, five basis functions were used (*n_w_* = 5), four of these were alpha functions (**G**_*i*_) with log-spaced time constants between *T*_1_ = 0.02 and *T*_4_ = 0.5 [see equation (10)] and one a constant filter implementing a bias term. These bases were chosen as they represent the range of time-scales expected to be required for DEAP plant compensation in a relatively compact way. A fixed matrix, estimated using SVD, was used to decorrelate and normalize parallel fiber signals to speed up learning. The learning rate *β* was chosen to give robust and stable learning on a time scale, which allowed tracking of variations in model parameters and a value of *β* = 4 was used in control experiments. The fixed matrix *Q* [see equation (8)] was calculated offline by estimating the brainstem output when there was no cerebellar contribution (*z* = 0). This brainstem output signal was then filtered through the bank of cerebellar alpha filters to give a matrix of filter output signals when there is no learning, Γ. An economy sized singular value decomposition was performed on the filter outputs (Γ) using the MATLAB function svd with “econ” option. This decomposition was then used to estimate *Q* as detailed in Section 2.1.

The DEAP actuators were comprised a thin, passive elastomeric film, sandwiched between two compliant electrodes. In response to an applied voltage, the electrodes squeeze the film in the thickness direction, resulting in biaxial expansion. In order to constrain the controlled variable to one degree of freedom, a spherical load was placed at the center of a circular DEAP and its motion in the vertical plane (i.e., vertical displacement) was measured (Figures 2A,B).

The DEAPs were made of acrylic elastomer (3 M VHB 4905) with an initial thickness of 0.5 mm. A conductive layer of carbon grease (MG chemicals) constitutes the capacitor plates. The elastomer was pre-stretched biaxially by 350% (where 100% was the unstretched length) prior to being fixed on a rigid Perspex frame with inner and outer diameters of 80 and 120 mm, respectively. The electrodes were brushed on both sides of the VHB membrane as circles with a diameter of approximately 35 mm. The load used during experiments was a sphere weighing 3 g.

A laser displacement sensor (Keyence LK-G152, repeatability – 0.02 mm) was used to measure the vertical movement of the mass sitting on a circular DEAP (Figure 2A). This signal was supplied to the input module of the CRio (Figure 2C). From the output module of the CRio, voltages were passed through a potentiometer (HA-151A HD Hokuto Denko) and amplified (EMCO F-121 high voltage module) with a ratio of 15 V:12 kV and applied to the DEAP.

We found that the DEAPs responded non-linearly over their full range of operation. In this investigation, we focused only on linear control over a sub-region of the DEAP dynamics, which allowed us to evaluate the MRAC scheme while retaining the power of linear systems analysis. Inputs to the DEAP were constrained to be small, so the response could be approximated as linear. This was done by limiting the displacement reference signal. The reference signal was low-pass filtered white noise with frequency range 0–1 Hz, and the amplitude constrained to 0.2–1. In total, the control experiment was repeated three times, with three separate DEAP actuators.

### 2.4 Model-based design of brainstem and reference model components

The cerebellar control scheme requires the *a priori* specification of the brainstem component, **B** and the reference model, **M** (Figure 2D). This section details how a simple model of the DEAP dynamics is estimated from input–output experiments, and how this model is used to inform design of the brainstem model **B** and reference model **M**.

The displacement responses of the DEAPs were modeled using a non-linear dynamic model with Hammerstein structure (static non-linear element followed by a linear dynamic element). This form of model was used as it is a simple, plausible way of accurately representing the DEAP dynamics. The response was modeled as

(20) $$a\dot{x}+x=\left\{\begin{array}{cc} \mathrm{bu}+c,\; & \text{if}\;u<e\\ \mathrm{bu}+c+d{\left(u-e\right)}^{2},\; & \text{otherwise}\\ \; \end{array}\right.$$

where *x* is the vertical displacement of the DEAP, *u* the voltage input (prior to amplification), and *a, b, c, d*, and *e* are the model parameters. The response is modeled as linear up to a point, then non-linear after this point. Model parameters were estimated by fitting the model to input–output data. In input–output experiments, the voltage input *u* was colored noise, containing frequencies between 0 and 1 Hz and ranging from 1.1 to 3.75 V. The algorithm used to estimate the model parameters was the trust region reflective method (this was implemented in MATLAB using the lsqnonlin function). Parameter estimates were initialized by setting *e* = 1 and using least squares. The dynamics of the DEAPs and their change over-time were measured by recording the displacement response to an applied voltage over a 30-min period. Initial model parameters were estimated by fitting to the first minute of data.

The linear response can also be described as a transfer function with one pole and no zeros, with a discrete offset (*c*/*b*) added to the input *u*,

(21) $$P\left(s\right)=\frac{b}{\mathrm{as}+1}.$$

A reference model (**M**) with the following form was used to ensure stability

(22) $$M\left(s\right)=\frac{1}{\tau s+1}$$

where *τ* = 0.1. A time constant of 0.1 s was used as this ensures that the controlled system responds reasonably quickly without causing stability problems (see section 2.2). The reference model is first order as the DEAP model has one pole and no zeros (see section 2.2).

Plant compensation is provided by a combination of a fixed brainstem controller **B** and adaptive cerebellar filter **C** [see equation (13)]. The fixed brainstem provides approximate feedforward control for the plant **B** and the adaptive cerebellar filter is used to fine tune this control and adapt to changes in the plant dynamics. The brainstem controller was designed to provide perfect compensation for an approximate plant $\hat{P}$, and is described using the following transfer function

(23) $$B\left(s\right)={\hat{P}}^{-1}\left(s\right)M\left(s\right)=\frac{a_{o}s+1}{b_{o}\left(\tau s+1\right)}$$

a discrete term (*b_d_* = − *c_o_*/*b_o_*) is added to the brainstem output. The parameters *a_o_*, *b_o_*, *c_o_* were based on the average parameters from plant models of six different DEAPs (*a_o_* = 0.087, *b_o_* = 0.28, *c_o_* = − 0.27 – averages from Table 2). Therefore, the brainstem controller provides compensation for the average DEAP model.

**Table 2:** **Estimated model parameters [equation (20)] for six different DEAP actuators**.

Actuator	*a*	*b*	*c*	*d*	*e*	VAF (%)
1	0.085	0.317	−0.196	0.788	2.320	95.0
2	0.068	0.225	−0.266	0.651	2.476	97.9
3	0.103	0.304	−0.318	1.242	2.517	97.8
4	0.094	0.511	−0.745	1.950	2.631	98.8
5	0.093	0.348	−0.327	1.023	2.552	98.5
6	0.077	−0.013	0.222	0.532	1.970	96.6

### 2.5 Summary of DEAP experiments

The DEAP actuators are not constructed to tight tolerances and are subjected to considerable variation in characteristics. To investigate the range of variation, we identified plant characteristics for six different DEAP actuators (actuators 1–6). The parameters of the fixed brainstem element were then estimated using the averaged parameter values. The fixed approximate feedforward controller is thus based on average manufacturing characteristics, and is not supposed to be designed separately for each actuator. The recurrently connected adaptive cerebellar filter was then applied to the control of two of the actuators used to estimate the fixed element (1 and 4) and to a third actuator not used in this estimate (actuator 7).

## 3 Results

This section presents results from applying the adaptive filter algorithm with MRAC extension to the displacement control of a DEAP actuator in one degree of freedom. We first present results on the open loop dynamics of the actuator, including estimated model parameters for six different DEAPs, which are used to design **B** and **M** for control. Results on the control performance of the extended cerebellar algorithm are then provided; the algorithms ability to control the DEAP to track a desired displacement signal over time is evaluated.

### 3.1 Open loop dynamics of dielectric electroactive polymer

**Figure 3:**
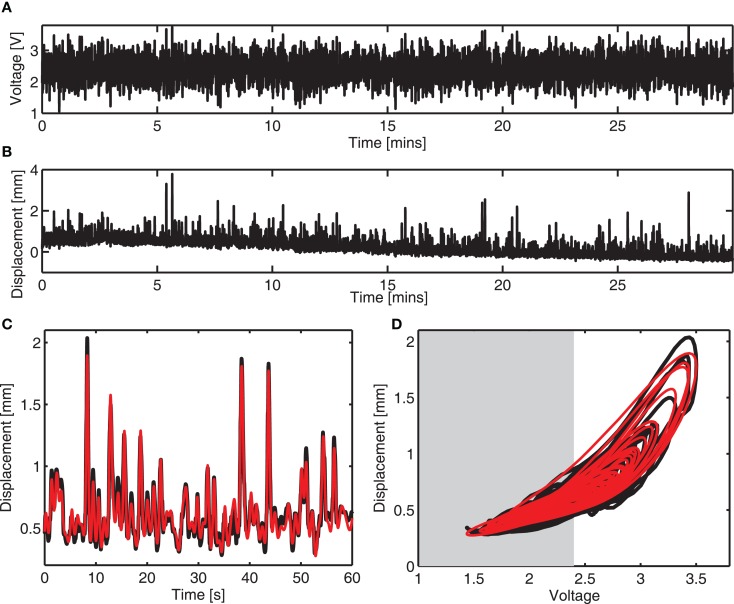
**Dynamic response and dynamic modeling of DEAP actuator**. **(A)** Voltage input prior to amplification. **(B)** Vertical displacement response of a mass on a circular DEAP during 30 min of actuation. **(C)** Measured (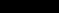) and modeled (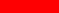) vertical displacement for one actuator in the time domain. **(D)** Measured (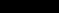) and modeled (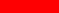) vertical displacement for one actuator. The range of response that can be modeled as linear is shaded.

A colored noise voltage was applied to the DEAPs over a 30-min period (Figure 3A), the corresponding displacement response of the DEAP actuator was found to change over time as shown in Figure 3B despite the input being similar over time. Model parameters, estimated as described in section 2.4, were obtained for six different actuators (Table 2). The variance accounted for (VAF) metric is also given for each actuator fit, this is a measure of the model fit quality to the observed data (VAF ≈ 100% implies the model fit is good). The model provides a reasonable description of the DEAP dynamics (Figures 3C,D) and demonstrates that total displacement response is non-linear but can be approximated as linear over a sub-region of the response. The region where the displacement response can be approximated as linear is highlighted in gray (Figure 3D).

### 3.2 Real-time experimental control

**Figure 4:**
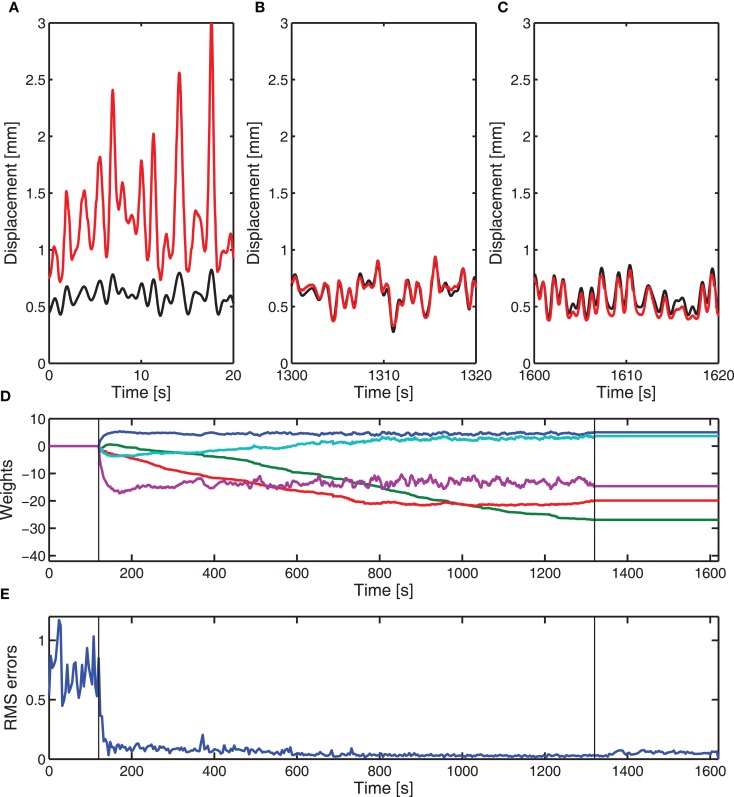
**Experimental adaptive control of DEAP (“actuator 7”) using cerebellar-inspired control**. Cerebellar weights are initially set to zero and the brainstem is an approximate fixed compensator. The vertical lines in **(D,E)** indicate when learning starts and stops. **(A)** Desired (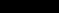) and actual (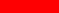) displacement of DEAP before learning. **(B)** Desired (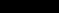) and actual (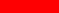) displacement response of DEAP during learning. **(C)** Desired (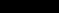) and actual (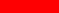) displacement response of DEAP after cerebellar learning has been stopped. **(D)** Estimated adaptive filter weights. **(E)** Windowed RMS errors during learning.

The cerebellar algorithm, with a fixed SVD-based parallel fiber optimization, was applied to the real-time control of a DEAP. The experimental results for one DEAP (“actuator 7”) that was manufactured to be similar, but a different actuator from the six used to estimate the brainstem model for one displacement tracking test are given in Figure 4. In this example, adaptation of the cerebellar weights (learning) started after 120 s from initial weights of zero, and was stopped after 1,320 s (indicated by vertical lines on Figures 4D,E). Before learning, the errors are large and during learning, the cerebellar weights adjust to minimize the errors. After learning is turned off, the weights no longer adapt to compensate for changes in the DEAP dynamics and the errors in displacement tracking gradually creep up.

**Figure 5:**
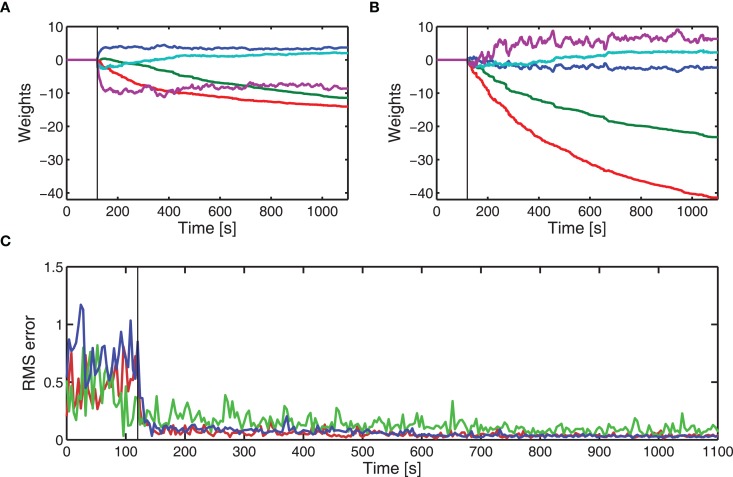
**Experimental adaptive control of different DEAPs using cerebellar-inspired control**. Vertical lines indicate when learning starts. **(A)** Learned weights when controlling actuator 1 (see Table 2). **(B)** Learned weights when controlling actuator 4 (see Table 2). **(C)** RMS errors over time during experiment for actuator 1 (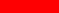), actuator 4 (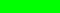), and actuator 7 (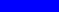).

Results from three different actuators are given in Figure 5. Two of these were actuators used in the estimate of the brainstem model, and the other a different actuator (see also Figure 4). For each DEAP, the weights adjust to minimize errors in the displacement tracking. Errors in tracking the displacement response for actuator 4 are slightly larger. The average applied voltages (prior to amplification) at the end of learning were 2.4, 2.9, and 2.3 V for actuators 1, 4, and 7, respectively. Inputs to actuator 4 are larger than those to the other actuators and above the range for which the behavior can be approximated as linear. The increased errors in tracking the displacement response for actuator 4 are likely to be caused by the actuator operating in its non-linear region during experiments.

**Figure 6:**
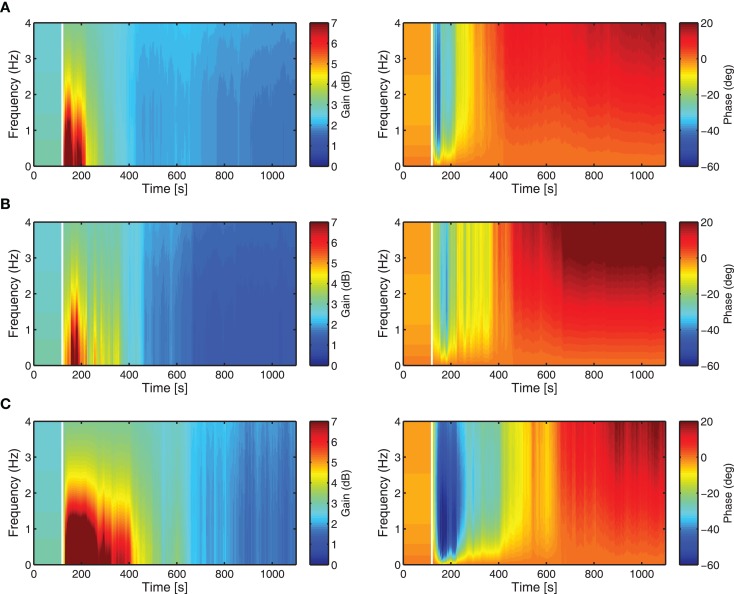
**Time-frequency response analysis of the cerebellar-brainstem controller K in terms of magnitude |K| (left) and phase ***∠***K (right)**. The evolution over time of the controller magnitude and phase shows how the adaptation of the cerebellar filter affects the controller dynamics over the time course of the DEAP experiments. Vertical white lines indicate when learning starts. **(A)** Actuator 1, **(B)** Actuator 4, **(C)** Actuator 7.

The adaptation of the controller **K** is analyzed here in terms of the time–frequency response, by plotting the variation in time of the magnitude, |**K**|, and phase, ∠**K** (this is essentially the Bode plot of **K**, re-evaluated at each time-step to show its dynamic evolution). These time–frequency response plots provide a more interpretable way of how change in adaptive filter weights (Figures 6A–C) affects the evolution through time of the overall controller **K**. Each controller has the same initialization (i.e., same brainstem and cerebellar filter weights initialized to zero) and shows a similar evolution over time in both the gain and phase responses. The frequency response reveals that the controller moves through a region of high gain in the early period of adaptation before converging to a smaller magnitude of gain, which is not obvious from the evolution of the adaptive filter weights.

## 4 Discussion

### 4.1 Control algorithm performance

The results demonstrated that the cerebellar algorithm with MRAC extension was able to accurately control the displacement of a type of artificial muscle, a DEAP actuator, in one degree of freedom. The results demonstrated stable learning and control performance across a set of three actuators. The dynamics of the DEAP actuators varied considerably across actuators and over time, likely to be due to manufacturing tolerances, creep, aging, and trauma. Despite these challenges, good control performance was achieved using the cerebellar algorithm without any initial tuning to the specific behavior of each actuator.

Prior to this investigation, we have shown that the cerebellar algorithm is able to control a system where the plant has equal poles and zeros by applying it to velocity control of an oculomotor plant (VOR) (Dean et al., 2002; Lenz et al., 2009). In this contribution, we have demonstrated that the algorithm can be extended to deal with displacement control of a strictly proper plant using MRAC. The MRAC-extended algorithm greatly increases the possible applications of the cerebellar algorithm for tasks in robotic control. In particular, the scheme opens up the possibility of controlling the force or impedance response in limb movements.

### 4.2 Cerebellar control

Biological systems have evolved successful control solutions that are robust, flexible, and applicable to a wide range of tasks. These control algorithms must learn *in situ* to adapt to any changes in the system and function during the learning process. Bioinspired control strategies may provide solutions to improve robotic control, especially within unstructured, complex, changing environments, and systems. An important step toward understanding the biological basis for motor control is an understanding of the algorithm implemented by the cerebellum. The uniformity of the cerebellar microcircuit suggests that a generic cerebellar algorithm is implemented in the control of many different tasks (Ito, 1984, 2008; Porrill et al., 2013). This leads to the idea of a “cerebellar chip,” where the function of each chip depends both on an internal algorithm and the external connections. The adaptive filter model is a plausible candidate for the internal algorithm [see Dean et al. (2010) for details] and here we propose a way of extending the algorithm to handle the control of strictly proper plants. This extension increases the potential applications of the cerebellar algorithm to robotic control.

Although the control scheme that we propose is biohybrid rather than biomimetic, it is interesting to consider whether the model reference approach could actually be implemented in biological systems. Only two substantive changes to the standard adaptive filter algorithm are required. The first is that errors in trajectory control must be calculated relative to the model output and not the reference trajectory. In fact, our knowledge of how exactly trajectories are specified in biological motor control, for example, the skeletomotor system, is so sparse that this question cannot yet be answered. The second change is that the learning rule requires a processed version of the parallel fiber signal. Such a processing stage is provided by the synaptic learning rule in which parallel fiber signals are filtered by an eligibility trace with a transfer function, which is approximately an alpha function as in equation (10) with *τ* ≈ 100 ms. If this matched the reference model order and time constants, it would supply two extra zeros, allowing control of plants with two more poles than zeros. An alternative and more general algorithm can also be designed in which it is the error signal rather than the parallel fiber signal, which is subjected to processing. At present, there is no clear biological evidence to favor either hypothesis.

### 4.3 Non-linear control

Here, we have focused on linear control over a sub-region of the DEAP dynamics. However, the dynamics of the DEAPs were non-linear over their full range of operation. Muscles also exhibit non-linearities and therefore the biological neuromuscular control system must also overcome non-linearities. The presented cerebellar algorithm could be extended to control non-linear systems by using a non-linear adaptive cerebellar filter, which includes non-linear basis, or by including an approximate plant linearization stage in the brainstem. Both of these methods fit well in the framework of the presented algorithm. The non-linear nature of DEAPs makes them a natural candidate for evaluating future non-linear cerebellar control algorithms.

### 4.4 Fast learning

**Figure 7:**
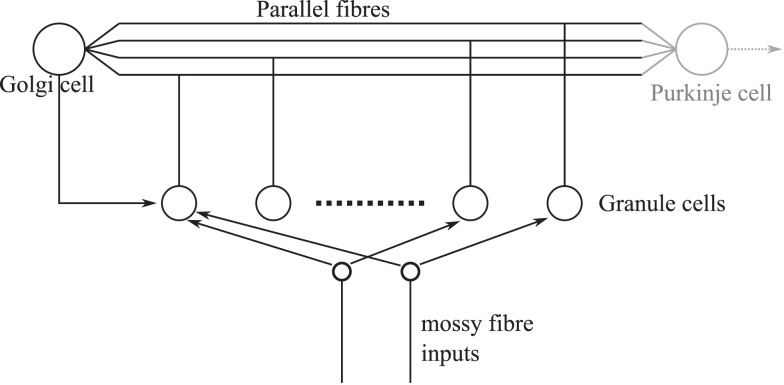
**Simplified granular layer of the cerebellum showing the recurrent connectivity between Golgi and granule cells**. The input from Golgi cells to granule cells is inhibitory. Golgi cells also receive a number of inputs from mossy fibers directly (not shown).

A fast learning transformation was used in the embodiment of the cerebellar algorithm in DEAP control to increase the rate of learning. For the given DEAP control task, this was achieved using an orthonormalization matrix, estimated from batch SVD decomposition of the expected motor command. It is pertinent to question whether such an orthonormalization procedure might occur in biology, in the cerebellum. If it were to do so, the orthonormalization of the input signal would likely occur in granular layer processing. The importance of the granular layer is highlighted by the fact that it contains the majority of the neurons in the brain (Herculano-Houzel, 2010). The granule layer has a recurrent architecture in which Golgi cells inhibit the granule cells (Figure 7). It has previously been suggested that plasticity in the granular network allows optimal bases to be learnt over time by a process of decorrelation via recurrent inhibition (Coenen et al., 2001). Interestingly, this inhibitory recurrent architecture also bears some resemblance to machine learning algorithms that perform a process of signal orthogonalization (Oja, 1989; Kung et al., 1994). Whether a similar process could occur in the granular layer of the cerebellum is an interesting area for future investigation.

## 5 Summary

The aim of this paper was to extend the adaptive filter model of the cerebellum to the control of strictly proper plants. This was achieved by augmenting the existing algorithm with a model reference adaptive control (MRAC) scheme. The performance of the biohybrid algorithm was evaluated by embodying it in the displacement control of an artificial muscle actuator, a dielectric electroactive polymer (DEAP). These DEAP actuators provide a good test of the algorithms performance as their dynamics vary over time and between actuators, meaning the controller must adapt to compensate for these variations. Results show that the extended cerebellar algorithm is able to accurately control the displacement response of several different DEAP actuators over time, and that MRAC provides a technical solution to extending the adaptive filter model to the control of strictly proper plants.

## Conflict of Interest Statement

The authors declare that the research was conducted in the absence of any commercial or financial relationships that could be construed as a potential conflict of interest.
